# Cell Death of *P. vivax* Blood Stages Occurs in Absence of Classical Apoptotic Events and Induces Eryptosis of Parasitized Host Cells

**DOI:** 10.3390/pathogens13080673

**Published:** 2024-08-09

**Authors:** Carolina Moreira Blanco, Hugo Amorim dos Santos de Souza, Priscilla da Costa Martins, Juliana Almeida-Silva, Ana Marcia Suarez-Fontes, Yury Oliveira Chaves, Marcos André Vannier-Santos, Lilian Rose Pratt-Riccio, Cláudio Tadeu Daniel-Ribeiro, Stefanie Costa Pinto Lopes, Paulo Renato Rivas Totino

**Affiliations:** 1 Laboratório de Pesquisa em Malária, Instituto Oswaldo Cruz, Fiocruz & Centro de Pesquisa, Diagnóstico e Treinamento em Malária (CPD-Mal), Secretaria de Vigilância em Saúde e Ambiente (SVSA), Ministério da Saúde, Rio de Janeiro 21040-360, Brazil; carolinamoreirablanco@gmail.com (C.M.B.); hugoamorims@gmail.com (H.A.d.S.d.S.); priscillamartins0502@gmail.com (P.d.C.M.); riccio@ioc.fiocruz.br (L.R.P.-R.); malaria@fiocruz.br (C.T.D.-R.); 2 Laboratório de Inovações em Terapia, Ensino e Bioprodutos, Instituto Oswaldo Cruz, Fiocruz, Rio de Janeiro 21040-360, Brazil; jualmeida@yahoo.com (J.A.-S.); marcos.vannier@ioc.fiocruz.br (M.A.V.-S.); 3 Instituto Leônidas e Maria Deane, Fiocruz Amazônia, Manaus 69057-070, Brazil; yurychaves@gmail.com (Y.O.C.); stefaniecplopes@gmail.com (S.C.P.L.); 4 Fundação de Medicina Tropical Dr. Heitor Vieira Dourado (FMT-HVD), Manaus 69040-000, Brazil

**Keywords:** *P. vivax*, cell death, metacaspase

## Abstract

Elucidation of pathways regulating parasite cell death is believed to contribute to identification of novel therapeutic targets for protozoan diseases, and in this context, apoptosis-like cell death has been reported in different groups of protozoa, in which metacaspases seem to play a role. In the genus *Plasmodium*, apoptotic markers have been detected in *P. falciparum* and *P. berghei*, and no study focusing on *P. vivax* cell death has been reported so far. In the present study, we investigated the susceptibility of *P. vivax* to undergo apoptotic cell death after incubating mature trophozoites with the classical apoptosis inducer staurosporine. As assessed by flow cytometry assays, staurosporine inhibited parasite intraerythrocytic development, which was accompanied by a decrease in cell viability, evidenced by reduced plasmodial mitochondrial activity. However, typical signs of apoptosis, such as DNA fragmentation, chromatin condensation, and nuclear segregation, were not detected in the parasites induced to cell death, and no significant alteration in metacaspase gene expression (*Pv*MCA1) was observed under cell death stimulus. Interestingly, dying parasites positively modulated cell death (eryptosis) of host erythrocytes, which was marked by externalization of phosphatidylserine and cell shrinkage. Our study shows for the time that *P. vivax* blood stages may not be susceptible to apoptosis-like processes, while they could trigger eryptosis of parasitized cells by undergoing cell death. Further studies are required to elucidate the cellular machinery involved in cell death of *P. vivax* parasites as well as in the modulation of host cell death.

## 1. Introduction

Malaria is a parasitic disease caused by protozoa of the genus *Plasmodium*, and is transmitted to humans by female *Anopheles* mosquitoes. This tropical disease remains one of the major public health problems in the world, accounting for 249 million cases and 608 thousand deaths worldwide in 2022 according to WHO estimates [1]. In this scenario, *P. vivax* is the most geographically widespread *Plasmodium* species and contributes significantly to the malaria burden outside Africa [1]. Moreover, *P. vivax* parasites resistant to chloroquine, which comprises the first-line treatment for vivax malaria in combination with primaquine, have been reported in many endemic areas, including Brazil, constituting a concern for malaria control programs [2,3].

In view of the risk of parasite chemoresistance and the consequent need for development of new antimalarial drugs, knowledge about the machinery of cell death in *Plasmodium* is of ultimate importance. Although the signaling pathways driving cell death in unicellular eukaryotes are unknown, hallmarks of apoptosis have been observed in different protozoa, such as *Toxoplasma gondii*, *Blastocystis hominis*, and *Trichomonas vaginalis* and species of the family Trypanosomatidae, under different stimuli, suggesting susceptibility to regulated cell death (RCD) processes [4,5,6,7]. Indeed, the use of caspase inhibitors argues for the existence of a cysteine protease-RCD in protozoa [8,9]. However, genes encoding caspases are absent in non-metazoan organisms, and the involvement of a cysteine protease family structurally related to caspases, named metacaspase, has been considered [9,10].

Metacaspases are expressed in the genus *Plasmodium* [11,12], and the capacity of *P. falciparum* metacaspase 1 (PfMCA1) to trigger apoptosis has been demonstrated in a yeast model [13]. In parallel, apoptotic events, including phosphatidylserine externalization, chromatin condensation, DNA fragmentation, and caspase-like activity, have been reported in *P. falciparum* blood stages and *P. berghei* ookinetes [14,15,16,17,18,19]. Nevertheless, in our earlier studies, blood stages of *P. falciparum* underwent a caspase-like independent cell death process marked by morphological evidence of autophagy [20], while a necrotic phenotype was detected by Porter and colleagues [21]. In *P. vivax*, although the genetic diversity of metacaspase 1 (PvMCA1) has been studied in different endemic countries [22,23,24], no focus has been given to the profile of cell death suffered by this *Plasmodium* species.

Therefore, in the present study, we investigated for the first time the susceptibility of *P. vivax* blood forms to undergo apoptotic events using a classical inducer of apoptosis, staurosporine.

## 2. Materials and Methods

### 2.1. P. vivax Parasites and Cell Death Induction

Clinical isolates of *P. vivax* were obtained from patients attending the Fundação de Medicina Tropical Doutor Heitor Vieira Dourado (FMT-HVD) in Manaus, Amazonas State, Brazil, according to the procedures approved by the Research Ethics Committee of FMT-HVD (CAAE 54234216.1.0000.0005 and 75894223.9.0000.0005). Patients presenting parasitemia higher than 500 parasites/µL blood with a predominance of asexual forms at the trophozoite stage (>60%), as evaluated by thick blood smears, were included and venous blood samples of each patient were collected by venipuncture with heparinized tubes.

Enrichment of parasites was performed as previously described [25]. Briefly, blood samples were centrifuged for plasma separation, leukocytes were depleted by filtration using columns of cellulose (Sigma-Aldrich, St. Louis, MO, USA), and blood pellets were diluted in RPMI 1640 medium (Sigma-Aldrich) to 10% hematocrit. Trophozoites were enriched in a 45% Percoll density gradient (GE-Healthcare, Uppsala, Sweden), washed with RPMI 1640 medium (Sigma), and then resuspended in IMDM (Gibco Industries, Big Cabin, OK, USA) supplemented with 20% of heat-inactivated human AB serum.

To evaluate the effect of staurosporine (Sigma) on the growth and cell death of *P. vivax*, enriched parasitized red blood cells (pRBCs; 1 × 10^6^ pRBCs/well) were incubated for 6–24 h in 96-well culture plates at 37 °C under an atmosphere of 5% CO_2_ (White Martins, Manaus, Brazil) in the presence or absence of staurosporine at concentrations (4 µM and 8 µM) described to induce cell death in different group of protozoa, including *P. falciparum* [4,5,7,9,20,21,26,27,28,29].

### 2.2. Evaluation of Parasite Growth Inhibition

Inhibition of parasite maturation was assessed by flow cytometry after 24 h using the Syto-16 nucleic acid stain (Thermo Fisher Scientific, Eugene, OR, USA) [30]. Briefly, pRBCs were washed by centrifugation (350× *g*, 5 min) in phosphate-buffered saline (PBS—0.15 mM NaCl, 2.5 mM KCl, 8 mM Na_2_HPO_4_ 7H_2_O, 1.5 mM KH_2_PO_4_), resuspended in PBS containing 100 µM Syto-16, and incubated at 37 °C for 40 min. After incubation, pRBCs were washed and resuspended in PBS, and then a minimum of 50,000 events were acquired on a FACSCanto II flow cytometer equipped with blue (488 nm) and red (633 nm) lasers (Becton Dickinson, Franklin Lakes, NJ, USA) using the FL1 channel (530/30 nm). Data were analyzed using FlowJo 10.0 software (Becton Dickinson) and are expressed as mean fluorescence intensity (MFI).

### 2.3. Viability and Apoptosis Assays

Parasite viability was assessed by measuring the mitochondrial transmembrane potential (ΔΨm) using rhodamine 123 (Thermo Fisher Scientific) as previously described [20], with modifications. After 24 h incubation, pRBCs were washed by centrifugation in PBS (350× *g*, 5 min) and incubated at 37 °C for 5 min in 1 µg/mL rhodamine. After removing rhodamine solution, pRBCs were washed and incubated for 30 min in PBS, resuspended in the same buffer, and analyzed in a flow cytometer. Cells were acquired using the FL1 channel, and data are expressed as mean fluorescence intensity (MFI).

The TUNEL (terminal deoxynucleotidyl transferase dUTP nick-end labeling) assay was used to verify parasite DNA integrity. Initially, pRBCs (5 × 10^6^) were treated with 0.1% saponin in PBS to obtain free parasites, as previously described [9], and then parasites were stained using an APO-DIRECT KIT (BD Pharmingen, San Diego, CA, USA) according to the manufacturer’s instructions. Briefly, parasites were fixed in 2% paraformaldehyde (1 h, 4 °C), washed twice by centrifugation in PBS (400× *g*, 5 min) and permeabilized with 70% ice-cold ethanol at −20 °C. Following removal of ethanol, parasites were incubated at 37 °C for 1 h in staining solution containing terminal deoxytransferase (TdT) and FITC-tagged deoxyuridine triphosphate nucleotides (FITC-dUTP). After incubation, parasites were rinsed twice with rinse buffer and finally analyzed in PBS by a flow cytometer using the FL1 channel. As a positive control, fixed/permeabilized free parasites were previously treated for 40 min at room temperature with 10 U/mL DNase I in DNase reaction buffer provided by the manufacturer (Thermo Fisher Scientific Life Sciences Solutions, CA, USA). Data are expressed as percentage of positive cells.

Externalization of phosphatidylserine was examined on the surface of pRBCs by annexin V assay (BD Pharmingen) in combination with Syto-16 staining. Syto-16 staining was performed as indicated above: pRBCs were resuspended in PBS containing 100 µM Syto-16, incubated at 37 °C for 40 min, and washed in PBS alone. Then, pRBCs were resuspended in 100 µL annexin-binding buffer containing 5 µL annexin V–APC and after 15 min incubation at room temperature, were fivefold diluted with annexin-binding buffer and lastly analyzed by flow cytometry. FL1 (Syto-16; 530/30 nm) and FL5 (annexin V-APC; 660/20 nm) channels were used, and data are expressed as a percentage of positive cells. Forward scatter (FSC) measurement (geometric mean) was additionally performed in both positive and negative pRBCs to estimate the difference in cell size.

A FACSCanto II flow cytometer and FlowJo 10.0 software (Becton Dickinson) were used for all experiments, and a minimum of 50,000 events was acquired. Supplementary Figures S1 and S2 show the gating strategy for the analysis of Syto-16 and rhodamine 123 staining (Figure S1) as well as annexin V/Syto-16 assays (Figure S2).

### 2.4. Analysis of PvMCA1 Gene Expression

Transcriptional expression of *Pv*MCA1 under cell death stimulation (staurosporine, 4 µM) was evaluated after 6 h and 18 h incubation. Parasites (1 × 10^6^) were stored in RNA stabilizing solution (Thermo Fisher Scientific Baltics, Vilnius, Lithuania) at −20 °C and total RNA was extracted using a PureLink RNA mini-Kit (Life Technologies Corporation, Austin, TX, USA), followed by treatment with DNase (Thermo Fisher Scientific Life Sciences Solutions) and reverse-transcription reaction using a high-capacity cDNA reverse transcription kit (Thermo Fisher Scientific Baltics), all according to the instructions of the manufacturers. cDNA obtained was quantified by a Qubit ssDNA Assay Kit (Molecular Probes, Eugene, OR, USA) and quantitative polymerase chain reaction (qPCR) was performed with a PowerUp SYBR Green Master Mix kit (Thermo Fisher Scientific Baltics) in a 7500 Real-Time PCR System (Applied Biosystems, Waltham, MA, USA). The qPCR conditions used were as follows: 2 min at 50 °C, followed by 2 min at 95 °C and 40 cycles of denaturation (95 °C/1 s) and annealing (60 °C/1 min). The specificity of amplification was verified through analysis of melting curves (95 °C/15 s, 60 °C/1 min, 95 °C/15 s). The reaction mixture consisted of 20 µL final volume containing 1XPowerUp SYBR Green Master Mix, primers (800 nM), cDNA (10 ng), and UltraPureDNase/RNase-Free Distilled Water (Thermo Fisher Scientific Life Sciences Solutions). All reactions were performed in duplicate and included DNase untreated samples and non-template negative controls. The *P. vivax* β-tubulin (Pvβ-T) housekeeping gene was assayed as internal control [31] and the following oligonucleotide primers were used: PvMCA1 (forward, 5′-ACCCCAGTGGACCACCAA-3′; reverse 5’-CACGAGGGTAAGTAACCCCA-3′); Pvβ-T (forward, 5′-CCAAGAATATGATGTGTGCAAGTG-3′; reverse, 5′-GGCGCAGGCGGTTAGG-3′). CT values were analyzed in the 7500 software (version 2.0.6), in which the fold change relative to the internal control was calculated through the 2^−∆∆ct^ formula [32].

### 2.5. Transmission Electron Microscopy

The morphology of the parasites induced to cell death was examined by transmission electron microscopy. Briefly, pRBCs were washed with PBS and fixed at 4 °C in 0.1 M sodium cacodylate buffer containing 2.5% glutaraldehyde. Then, samples were post-fixed in 1% osmium tetroxide, 5 mM calcium chloride, and 0.8% potassium ferricyanide in sodium cacodylate buffer, followed by dehydrating in acetone. Samples were embedded in Polybed epoxy resin (Polysciences Inc., Warrington, PA, USA), and after polymerization at 60 °C, were sectioned on an ultramicrotome. The sections were collected on copper grids, counterstained with 3% lead citrate and 5% uranyl acetate in water, and observed under a transmission electron microscope (JEOL-JEM-1011; JEOL USA Inc., Peabody, MA, USA).

### 2.6. Statistical Analysis

Statistical analyses were performed using GraphPad Prism 5.0 software (San Diego, CA, USA) and differences were tested by *t* test or one-way ANOVA with Dunnett’s post-test. A *p*-value of <0.05 was considered statistically significant.

## 3. Results and Discussion

**Figure 1:**
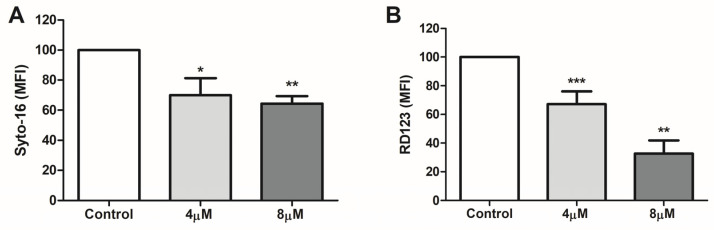
Inhibition of *P. vivax*-blood stage development by staurosporine. Percoll-enriched trophozoites were incubated for 24 h in the absence (control) or presence of staurosporine (4 µM and 8 µM), and then parasite development (**A**) and viability (**B**) were measured by flow cytometry assays using Syto-16 and rhodamine 123 (RD123), respectively. Data are mean fluorescence intensity (MFI) of gated positive cells in which DNA content (Syto-16; **A**) and mitochondrial activity (RD123; **B**) were determined. Results are presented as means ± standard error (SE) of five independent experiments, normalized to percentage of non-treated parasites (control: 100%). *: *p* < 0.05; **: *p* < 0.01; ***: *p* < 0.001 compared with control.

To study the susceptibility of *P. vivax* blood stages to apoptosis-like cell death, we initially evaluated the potential of staurosporine to inhibit parasite development after 24 h incubation of mature trophozoites, which are the most metabolic stages of the *Plasmodium* intraerythrocytic cycle [33], constituting ideal targets for our pioneering investigation on *P. vivax* cell death. Staurosporine is a broad-spectrum protein kinase inhibitor that has been widely used as an inducer of apoptosis in metazoan cells [34,35], presenting also the capacity to both inhibit cell growth and trigger apoptotic events in different groups of protozoa [7,36,37]. Indeed, protein kinases are key components of cell signaling and regulation found ubiquitously across the domains of life [38], and their blockage in *Plasmodium* has been shown to arrest the progression of intraerythrocytic development in a dose-dependent manner, with the appearance of abnormal morphological forms [39,40]. Therefore, it was not surprising that staurosporine also significantly inhibited the maturation of *P. vivax* trophozoites, as herein evidenced by the diminished DNA content in flow cytometry assays compared to non-treated parasites (Figure 1A).

Due to the obstacles to evaluating parasite viability applying classical cell membrane permeability dyes, as *Plasmodium* parasites develop inside erythrocytes surrounded by two additional membranes (host cell membrane and parasitophorous vacuole membrane), the mitochondrial membrane potential (ΔΨm), which is disrupted in dying cells, was measured as an indicator of plasmodial viability [20,41,42,43,44]. Some studies have reported that the mechanism of staurosporine-induced cell death involves increased ROS generation [45,46,47] that in turn results in mitochondrial damage and loss of ΔΨm [48]. In accordance with this pathway, disruption of ΔΨm was observed after induction of both apoptotic and necrotic cell death in *P. falciparum* trophozoites by staurosporine [9,21], a mitochondrial alteration that was also recorded by us in *P. vivax* and that seemed to be dose-dependent (Figure 1B).

**Figure 2:**
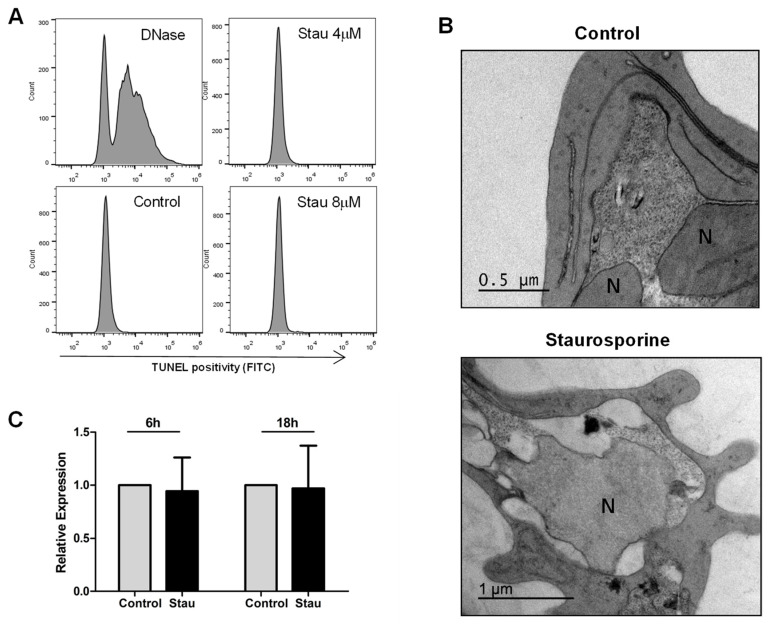
Staurosporine induces non-apoptotic cell death in *P. vivax* parasites. Percoll-enriched trophozoites were incubated for 6–24 h in the absence (control) or presence of staurosporine (Stau; 4 µM or 8 µM) and then apoptotic events were examined. (**A**) Cytometric analysis of TUNEL assay after 24 h incubation using DNase-treated parasites as positive control. (**B**) Representative electron micrographs of staurosporine-induced cell death analysis. Control parasites showed normal morphology, with regular nuclei (N), preserved cytoplasm, and close juxtaposition of plasma and parasitophorous vacuole membranes. Staurosporine-treated parasites presented marked retraction and loss of cytoplasmic content as well as irregular nucleus shape, but absence of detectable chromatin condensation and nuclear segregation. (**C**) Gene expression levels of PvMCA1 under cell death stimulus (4 µM staurosporine), as evaluated by qPCR with *P. vivax* β-tubulin gene as endogenous control. Results are presented as means ± standard error of three independent experiments, and no significant difference was observed between control and staurosporine-treated parasites.

Although the occurrence of RCD processes in malaria parasites is still a matter of debate, studies on *P. berghei* have demonstrated that ookinetes suffering spontaneous or induced cell death display typical features of apoptosis, including loss of mitochondrial membrane potential, exposure of phosphatidylserine, chromatin condensation, DNA fragmentation, and caspase-like activity [49,50,51]. In *P. falciparum*, cell death has been mainly explored in blood stages under different in vitro conditions, and apoptosis-like cell death has been fundamentally reported through the detection of DNA fragmentation, as also shown by Arambage and colleagues (2009) [50] for *P. falciparum* ookinetes. In our study, however, no expressive levels of DNA fragmentation (˂1.5% parasites) were observed in the *P. vivax* blood stage after incubation, either in the presence or absence of the cell death inducer staurosporine (Figure 2A).

Similar levels of TUNEL-positive parasites and even absence of DNA fragmentation [21,52] have already been found in *P. falciparum* blood forms stimulated to cell death by different inducers, including staurosporine [20,53], and additional ultrastructural analysis of dying parasites showed no signs of classical apoptotic processes, i.e., early chromatin condensation or nuclear segregation [9,20,21,53,54,55], arguing against deflagration of apoptotic cell death in blood forms of *P. falciparum*. Indeed, electron microscopy examination has not been routinely applied to the evaluation of *P. falciparum* apoptosis [14,15,16,17,18,19], and in agreement with the abovementioned works, we observed that staurosporine-induced cell death in *P. vivax* parasites was not characterized by typical ultrastructural alterations of apoptosis, but by a degenerative cell process with loss of cytoplasmic content (Figure 2B), in which the involvement of parasite digestive vacuoles must still be investigated [55,56]. Thus, it is possible that the TUNEL positivity observed by us resulted from non-specific cell degeneration related to late steps of cell death, as occurs in necrosis, or alternatively that it reflects other cell processes in which the 3’-hydroxyl termini of DNA strands become exposed, as during DNA repair and gene transcription [57]. Above all, it must be considered that the ultrastructural morphology of staurosporine-treated *P. vivax* herein observed (Figure 2B) was very similar to the necrotic *P. falciparum* trophozoites induced by staurosporine in a study conducted by Porter and colleagues [21]. In such study, dying parasites with degradation of cytoplasm and absence of typical apoptotic signs were also found under incubation of *P. falciparum* with heat (40 °C) or chloroquine, supporting necrosis being a common type of cell death in *Plasmodium*.

It has already been described that cell death in malaria parasites is a Ca^2+^-dependent and transcriptionally regulated process involving the activity of cysteine proteases [9,10,56]. Since caspases are absent in unicellular eukaryotes, the members of the metacaspase family have been implicated in the execution of cell death in protists [58], and in this context, the ability of *P. falciparum* metacaspase 1 (*Pf*MCA1) to trigger cell death machinery was elegantly demonstrated in a yeast cell model [13]. Therefore, to obtain some insight into the *P. vivax* death pathway, we evaluated the expression of the *pvmca1* gene under cell death stimulation, and no significant change was detected comparing non-treated parasites and parasites treated with staurosporine for 6 h and 18 h (Figure 2C), suggesting that *Pv*MCA1 is not related to cell death signaling in the *P. vivax* blood stage, at least at the transcriptional level. Of note, a role of metacaspase 2 in the progression of the parasite life cycle was recently shown in *P. falciparum* and *P. berghei* [59], and in the fungi models *Saccharomyces cerevisiae* and *Ustilago maydis*, metacaspases played a role in the clearance of intracellular insoluble protein aggregates [60,61], raising the possibility of a non-death function of *Pv*MCA1.

**Figure 3:**
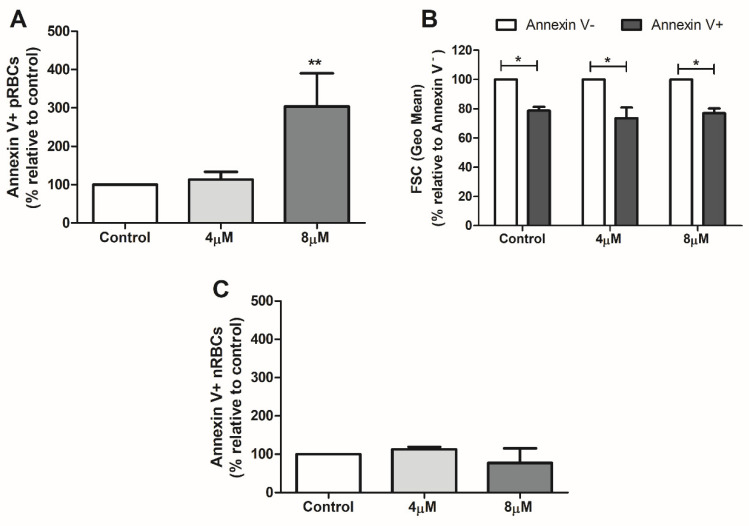
Staurosporine-induced parasite cell death positively modulates eryptosis of parasitized red blood cells (pRBCs). Percoll-enriched trophozoites were induced to cell death for 24 h with staurosporine (4 µM and 8 µM), and eryptosis of pRBCs (Syto-16-positive cells) and nRBCs (Syto-16-negative cells) was evaluated by annexin V staining (**A**,**C**) and cell size measurement (**B,** pRBCs) in flow cytometry analysis. (**A**) Levels of phosphatidylserine-exposed pRBCs (annexin V+), normalized to percentage of non-treated parasites (control: 100%). (**B**) Cell size of eryptotic pRBCs (annexin V+) compared to non-eryptotic pRBCs (annexin V-; 100%). (**C**) Levels of phosphatidylserine-exposed nRBCs (annexin V+), normalized to percentage of non-treated parasites (control: 100%). Data are presented as means ± standard error of four independent experiments. *: *p* < 0.05; **: *p* < 0.01, compared with annexin V- (**B**) and control (**A**), respectively.

Lastly, we examined the impact of dying parasites on the eryptosis of parasitized red blood cells. Eryptosis is an RCD process occurring in red blood cells that can be triggered by diverse stimuli and is characterized by apoptotic hallmarks of nucleated cells, including phosphatidylserine externalization and cell shrinkage [62]. In malaria, increased levels of eryptosis have been described in both parasitized and non-parasitized RBCs, and the contribution of such a cell phenomenon to the pathogenesis of the disease has been considered [30,63]. Interestingly, we observed that induction of cell death in *P. vivax* trophozoites by staurosporine also positively modulated eryptosis of pRBCs, which was evidenced using annexin V staining and cell size measurement (Figure 3A,B), while no significant alteration in the levels of annexin V-positive nRBCs was detected (Figure 3C).

This observation is in agreement with the well-known capacity of intracellular parasites to manipulate the host cell death pathways [64,65], and most importantly brings additional insight into the parasite–host relationship, in which dying parasites seem to retain the ability to trigger cell death of their host cells, indicating the existence of cross talk between the cell death signaling of these entities. Although the relevance and mechanisms whereby this interconnection takes place are unknown, one can hypothesize that induction of apoptosis/eryptosis in host cells by intracellular dying parasites can operate as an altruistic strategy of immune system evasion, considering that cells undergoing apoptotic processes are endowed with anti-inflammatory properties [66]. Further studies addressing this topic are necessary to better clarify the potential of dying parasites to modulate the host cell death machinery.

## Data Availability

Data are contained within the article.

## Supplementary Materials

The following supporting information can be downloaded at: https://www.mdpi.com/article/10.3390/pathogens13080673/s1, Figure S1: Representative gating strategy for analysis of parasite growth inhibition and viability, using Syto-16 and rhodamine 123 staining, respectively. Single cells were gated and the RBC population was identified by morphology parameters (SSC/FSC). Parasitized red blood cells (pRBC) were selected based on Syto-16 (A) or rhodamine 123 (B) positivity and then, the mean fluorescence intensity of each dye was estimated as shown in the histograms. Solid lines: control (non-treated parasites); dashed lines: staurosporine-treated parasites.; Figure S2: Gating strategy for the analysis of phosphatidylserine externalization in both non-parasitized (nRBC) and parasitized (pRBC) red blood cells. Single cells were gated, and the RBC population was identified by morphology parameters (SSC/FSC). Syto-16 positive (pRBC) and negative (nRBC) events were then analyzed for annexin V positivity.
