# Supplementary material for: Cell Death of P. vivax Blood Stages Occurs in Absence of Classical Apoptotic Events and Induces Eryptosis of Parasitized Host Cells
Source: Pathogens. 2024 Aug 9;13(8):673. doi: 10.3390/pathogens13080673 (PMC11357032; doi:10.3390/pathogens13080673)
Supplement: Supplementary file 1 [file pathogens-13-00673-s001.zip › Figure S1.pdf]

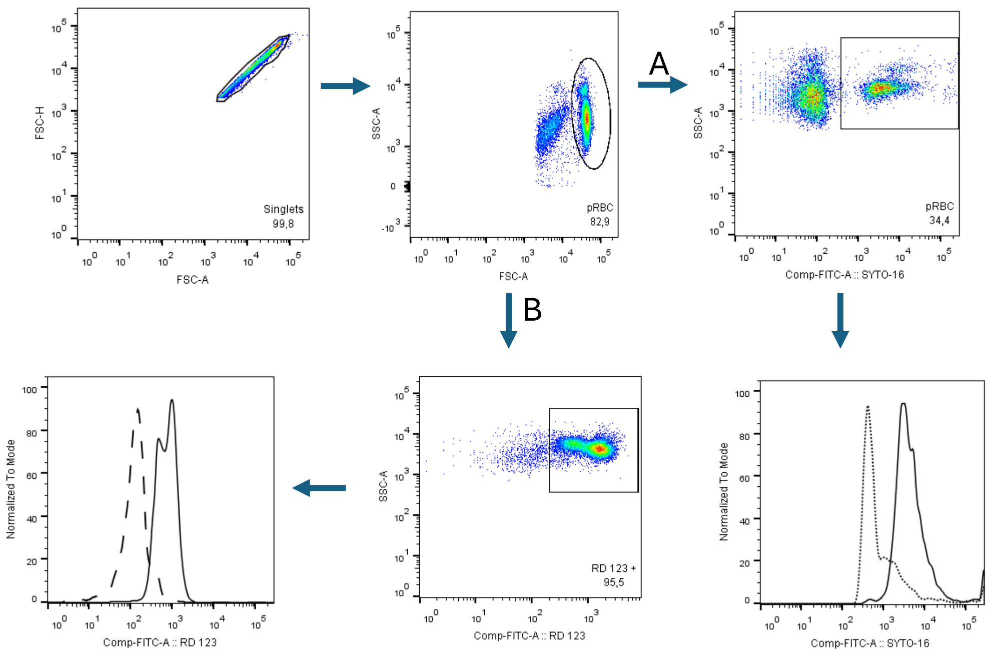

Supplementary figure S1. Representative gating strategy for analysis of parasite growth inhibition and viability, using Syto-16 and rhodamine 123 staining, respectively. Single cells were gated and the RBC population was identified by morphology parameters (SSC/FSC). Parasitized red blood cells (pRBC) were selected based on Syto-16 (**A**) or rhodamine 123 (**B**) positivity and then, the mean fluorescence intensity of each dye was estimated as shown in the histograms. Solid lines: control (non-treated parasites); dashed lines: staurosporine-treated parasites.
