# Supplementary material for: Cell Death of P. vivax Blood Stages Occurs in Absence of Classical Apoptotic Events and Induces Eryptosis of Parasitized Host Cells
Source: Pathogens. 2024 Aug 9;13(8):673. doi: 10.3390/pathogens13080673 (PMC11357032; doi:10.3390/pathogens13080673)
Supplement: Supplementary file 1 [file pathogens-13-00673-s001.zip › Figure S2.pdf]

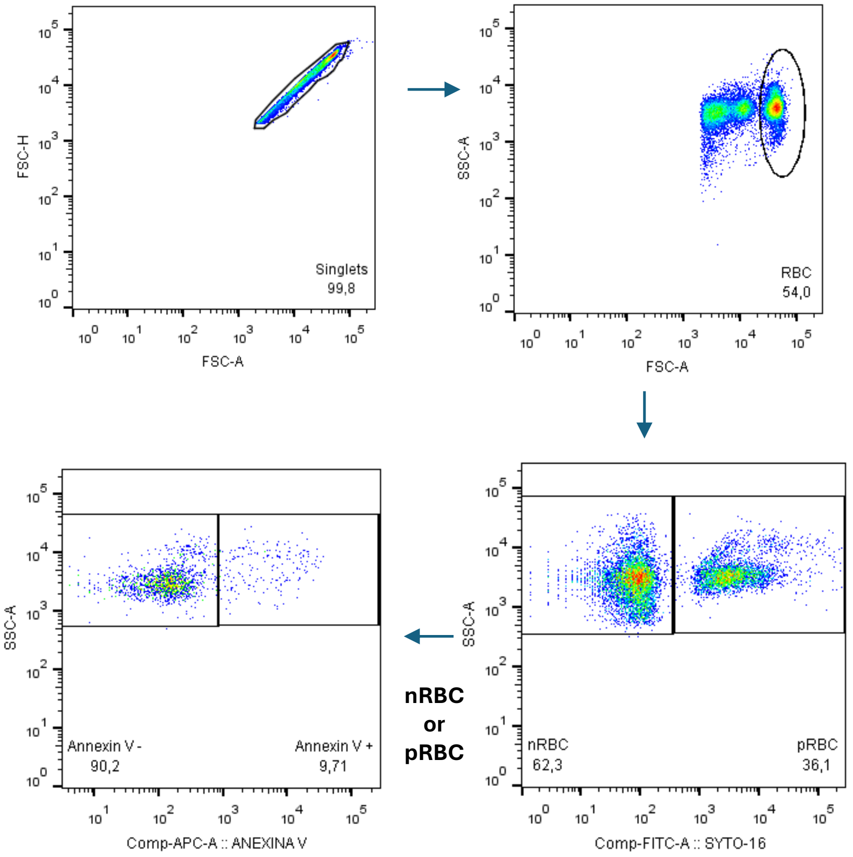

Supplementary figure S2. Gating strategy for the analysis of phosphatidylserine externalization in both non-parasitized (nRBC) and parasitized (pRBC) red blood cells. Single cells were gated, and the RBC population was identified by morphology parameters (SSC/FSC). Syto-16 positive (pRBC) and negative (nRBC) events were then analyzed for annexin V positivity.
